# Measuring Patient Experience to Promote Health Equity: Development and Validation of the Patient Experience of Social Needs Screening Questionnaire

**DOI:** 10.1177/24731242251390227

**Published:** 2025-11-10

**Authors:** Christopher K. Rogers, Manisha Parulekar

**Affiliations:** ^1^ Hackensack Meridian Hackensack University Medical Center, Hackensack, New Jersey, USA.; ^2^ Division of Geriatrics, Hackensack Meridian School of Medicine, Hackensack Meridian Hackensack University Medical Center, Hackensack, New Jersey, USA.

**Keywords:** social determinants of health, health equity, social needs screening, psychometrics, patient experience

## Abstract

**Introduction:**

Screening for social determinants of health (SDOH) is a critical strategy to advance health equity among underserved and vulnerable populations. Despite growing adoption, few validated, quantitative tools exist to measure patients’ experiences with screening tools and clinical workflows. The Patient Experience of Social Needs Screening (PESNS) questionnaire was developed to fill this gap.

**Objective:**

To develop and validate a 9-item PESNS questionnaire to measure patient perceptions of SDOH screening tools and workflows in ambulatory, inpatient, and emergency department settings, with a focus on supporting health equity and patient-centered care.

**Methods:**

A comprehensive literature review informed the development of 27 constructs. Using a modified Delphi process with eight experts, 29 preliminary items were refined across four rounds, resulting in a final 9-item questionnaire. Spanish translation followed best practices, including cognitive testing with six patients. The PESNS questionnaire was administered via telephone to 262 Medicare and Medicaid patients. Principal component analysis, exploratory factor analysis, and polychoric correlations assessed construct validity. Internal consistency was measured using Cronbach’s α.

**Results:**

Two components emerged—Enhancing User Experience Essentials and Patient-Centered User Experience—accounting for 60% of variance. Cronbach’s α was 0.841 overall, with factors 1 and 2 scoring 0.790 and 0.848, respectively. All items showed acceptable correlations (0.438–0.669), supporting scale reliability and construct validity. Component 1 captures the usability and effectiveness of the PESNS questionnaire, and Component 2 assesses privacy and environmental comfort. These dimensions align with health equity goals by highlighting patient-centered barriers and facilitators to SDOH screening.

**Conclusion:** The PESNS questionnaire offers a reliable, valid, and equity-focused tool to assess patient experience with SDOH screening. Its adoption can inform quality improvement and support more inclusive and effective health care delivery.

## Introduction

In recent years, patient screening for social determinants of health (SDOH), also known as health-related social needs (HRSN), has emerged as a critical strategy for health care providers to advance health equity among underserved populations by identifying social needs, addressing health disparities, and implementing evidence-based interventions.^1^ Several studies have reported validated processes and questionnaires for SDOH screening.^2–5^ Qualitative research highlights patient trust, confidentiality, and culturally sensitive approaches as key factors influencing screening success,^6^ and other studies explore provider challenges in integrating SDOH screening into workflows.^7^

Although SDOH screening interventions have been assessed across diverse settings, few validated, quantitative tools exist to measure patients’ experiences with screening tools and workflows in health care. HRSN screening is central to initiatives such as the Centers for Medicare & Medicaid Services (CMS) Accountable Health Communities (AHC) Model, aiming to improve outcomes and decrease costs by systematically addressing patients’ social needs.^6,8^ Despite growing adoption, major gaps remain in understanding patient perceptions, which critically influence trust, participation, and program success.^9^

While not yet mandated in all clinical encounters, SDOH screening is increasingly required, particularly in Medicare and Medicaid populations, with CMS mandating inpatient SDOH screening in 2024.^10^ Yet, screening rates vary widely across health care settings, with only 16.7% of primary care visits and 0.2% of emergency department (ED) visits including SDOH screening.^11^ This indicates that a significant portion of patients are not routinely assessed for SDOH, potentially missing out on crucial social needs evaluations and interventions. This gap underscores the need for quantitative tools that evaluate not only the screening process but also patient experiences with social needs screening tools to identify the barriers and facilitators to participation.

### Specific aims

The 9-item Patient Experience of Social Needs Screening (PESNS) questionnaire was developed to advance health equity by measuring patients’ perceptions of SDOH screening tools and clinical workflows across ambulatory, inpatient, and ED settings. Validated at a suburban academic and research hospital, the PESNS scale supports health care quality improvement efforts to optimize social needs screening and enhance patient-centered care.

### Importance of the construct: Patients’ experience with the HRSN screening tool

The development of the PESNS scale is grounded in the growing emphasis on addressing SDOH in clinical care.^1^ Patients’ experiences with SDOH screening involve perceptions of usability, relevance, comfort, privacy, and satisfaction, which directly influence screening uptake and effectiveness.^6,7,9^ Negative experiences, such as stigma or poorly designed tools, can hinder participation.^12–14^ The PESNS scale systematically captures these facets, offering health care systems actionable insights to refine screening practices, enhance patient-centered care, and advance health equity. The construct of “patients’ experience with HRSN screening” is inherently complex, as it integrates multiple dimensions of perceptions of the screening tool’s clarity, relevance, accessibility, and the comfort and privacy of the environment. Measuring this construct helps evaluate the patient-centeredness of SDOH screening processes and informs strategies to improve social needs identification and advance health equity.

## Methods

### PESNS questionnaire development

**FIG. 1.**
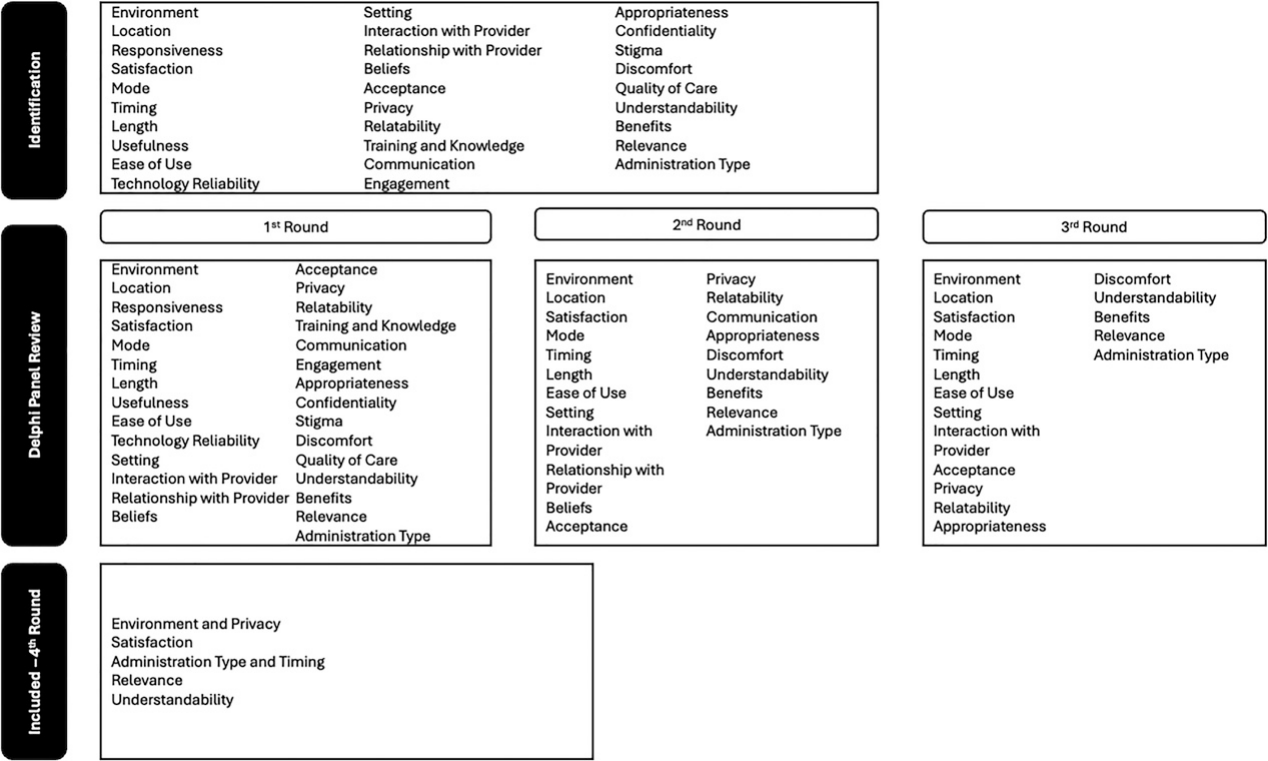
Flow diagram of constructs used for PESNS questionnaire development. PESNS, Patient Experience of Social Needs Screening.

The final PESNS questionnaire was developed using a modified Delphi method. It is a 9-item, approximately 250-word questionnaire designed to assess patients’ experiences with SDOH screening tools and clinical workflows in health care settings. The principal researcher (C.K.R.) conducted a comprehensive literature review to identify constructs for initial questionnaire development. Figure 1 shows the initial 27 constructs identified and the 9 constructs included in the final questionnaire after the four-round Delphi process. The full PESNS questionnaire is available in the Supplementary Material.

### Literature review search strategy

The comprehensive literature review identified 27 constructs foundational to PESNS development. Searches across PubMed, CINAHL, PsycINFO, Scopus, and Web of Science focused on patient experiences with HRSN screening tools, including usability, relevance, satisfaction, and privacy. Additional searches reviewed validated tools such as the AHC Model and PRAPARE.^15^ Methodological terms such as “Delphi method,” “cognitive testing,” and “psychometric analysis” ensured the inclusion of studies supporting robust, evidence-based questionnaire development.

### Initial questionnaire development

Using best practices for questionnaire design,^16–17^ original questions were developed to align with the 27 constructs, ensuring the coverage of specific dimensions of patient perceptions with social needs screening. Two constructs—“technology reliability” and “training and knowledge”—required two separate questions each due to their complexity, resulting in 29 preliminary questions. Constructs and questions were carefully crafted to capture nuanced patient experiences across diverse health care settings. Subsequent cognitive testing and expert panel reviews refined the items for relevance and clarity.

### Modified Delphi process and item refinement

Eight expert stakeholders, including clinicians and SDOH researchers, were selected to participate in a modified Delphi process to refine the PESNS questionnaire.^18,19^ Experts participated in four feedback rounds. Items were designed to be brief, simple, culturally competent, and patient-centered.

In round 1, panelists reviewed the 29 preliminary items using an open-ended format. They emphasized focusing on patient-centered content rather than on system-level processes and recommended simplifying technical terms (e.g., replacing “health care infrastructure” with “screening location”) and structuring Likert scales consistently.

In round 2, experts rated each item using a 5-point scale and provided comments. As 80% consensus was not reached, items were revised for clarity, relevance, and elimination of redundancy. In round 3, experts re-rated the revised items, and in round 4, final ratings and minority views were reviewed. After the four rounds, 18 items were removed, resulting in the final 9-item PESNS questionnaire. The remaining items were clear, highly relevant, and aligned with the intended constructs.

### Spanish translation and cognitive testing with patients

To ensure linguistic and conceptual equivalence, a rigorous translation process was employed.^20–22^ A bilingual research assistant forward-translated the English questionnaire, and a second independent assistant back-translated it while documenting challenges. Flaherty’s 3-point scale was used to assess translation quality.^23,24^ Three bilingual Delphi experts reviewed both versions, resolving discrepancies through consensus.

Face validity was confirmed by a team of bilingual, culturally competent experts and research assistants. The finalized Spanish PESNS questionnaire underwent cognitive testing with six Spanish-speaking adults aged 42–68 years.^25^ A bilingual research assistant administered the questionnaire via telephone, pausing after each section to assess comprehension and address concerns. Minor rephrasing was needed only for the introduction. Based on feedback and expert analysis, a fully reconciled Spanish version was finalized, ensuring the instrument’s validity and reliability across languages and cultural contexts.

### Survey population and administration

The PESNS questionnaire was telephone-administered in English and Spanish within 6 weeks post-visit by four trained research assistants (three involved in the Spanish translation and cognitive testing) using a principal researcher-developed interview protocol. A random sample of 262 Medicare and Medicaid patients screened for SDOH at Hackensack University Medical Center completed the PESNS questionnaire, with a 69.6% response rate (262/376). Guardians of Medicaid-enrolled children who completed the AHC HRSN Screening Tool also participated. Patients were randomly selected from ambulatory, inpatient, or ED settings and completed the screening via self- or interviewer-administration using an iPad/tablet, a hard copy, or a computer electronic health record. Verbal consent and age verification were obtained for all participants. Patients were informed that the call aimed to assess their perceptions of the CMS AHC HRSN Screening Tool and process. Background health questions were collected but excluded from factor analysis.

This study received Institutional Review Board approval from Hackensack University Medical Center (Pro2017-0396). Verbal informed consent was obtained over the telephone before participation and documented by the interviewer, with forms securely stored alongside participants’ questionnaire responses.

### Statistical analysis

Central tendencies were reported as means for normally distributed data. A nonparametric Kruskal–Wallis test was conducted to determine differences across groups (e.g., administration method, setting, and general health). For validation, both principal component analysis (PCA) and exploratory factor analysis (EFA) were performed, as the combined use of these methods provides complementary insights into the underlying structure of the data.^26^ PCA with varimax rotation, the Kaiser–Meyer–Olkin (KMO) measure, and Bartlett’s test of sphericity assessed the nine PESNS items’ structure, retaining factors with eigenvalues >1.0 and item loadings >0.50.^27^

Given the polytomous response options, structural validity was further assessed using polychoric correlations and EFA.^28,29^ Parallel analysis and scree plots determined the number of factors,^30^ preserving factors where real-data eigenvalues surpassed random-data eigenvalues.^31^ Parallel analysis for EFA using principal axis/common factor analysis (PA/CFA) on the nine items was conducted using the estimated polychoric correlation matrix.^29^ An oblique rotation evaluated correlations among components.^32^ A coefficient threshold of 0.40 was used for factor loadings, with *k* = 4 for rotation.^33,34^

Internal consistency was assessed using Cronbach’s α to evaluate the reliability of the PESNS items within each component, with α values between 0.70 and 0.80 considered acceptable.^35^ Corrected item–total correlations were also examined to identify any items that correlated poorly with the overall scale (i.e., correlations <0.30).^35^

## Results

A total of 262 (141 Medicare and 116 Medicaid) individuals completed the telephone-administered PESNS questionnaire, including 127 Spanish speakers, with 98% completing all nine questions. Respondents were mostly female (66%) and Hispanic or Latino (49%), with 51.5% aged 65 years or older. Most (66.5%) had at least a high school diploma. Sixty-two percent completed an interviewer-administered AHC HRSN screening during their encounter, and 38% self-administered. Seventy-six percent reported good to excellent health. The social needs reported included food insecurity (25%), transportation problems (16%), housing instability (11%), utility needs (12%), and safety concerns (4%).

Table 1 reports the item means, standard deviations, and response patterns. Cronbach’s α for the nine items was 0.841, with Factors 1 and 2 at 0.790 and 0.848, respectively, indicating high reliability. Table 2 shows item correlations ranging from 0.438 to 0.669, demonstrating very good discrimination. Satisfaction, privacy, and setting comfort showed the highest correlations, whereas understandability and the screening tool length had lower correlations.

**Table 1.** Descriptive Statistics of PESNS Items by Component (Cronbach’s **α**)

PESNS question (9-item Cronbach’s α = 0.841)	*n*	Mean	SD	Response category	Proportion
Component 1: Enhancing User Experience Essentials (α = 0.790)					
Relevance					
How would you rate the screening tool’s ability to identify your health-related social needs?	259	4.16	0.810	1—Very poor	0.015
				2—Poor	0.004
				3—Acceptable	0.154
				4—Good	0.456
				5—Very Good	0.371
How appropriate did you find the questions on the screening tool?	259	4.23	0.737	1—Absolutely inappropriate	0.008
				2—Inappropriate	0.019
				3—Neutral	0.077
				4—Appropriate	0.525
				5—Absolutely appropriate	0.371
Understandability					
The understandability of the screening tool was:	256	4.41	0.678	1—Very difficult to understand	0.000
				2—Difficult to understand	0.008
				3—Neutral	0.085
				4—Easy to understand	0.398
				5—Very easy to understand	0.510
Administration type and timing					
How satisfied were you with the mode in which you completed the screening tool?	260	4.40	0.676	1—Very dissatisfied	0.000
				2—Dissatisfied	0.019
				3—Neutral	0.050
				4—Satisfied	0.442
				5—Very satisfied	0.488
How would you describe the length of the screening tool?	260	3.94	0.926	1—Very long	0.004
				2—Long	0.050
				3—Neutral	0.285
				4—Short	0.323
				5—Very short	0.338
Satisfaction					
Overall, how satisfied were you with the screening tool process?	258	4.37	0.764	1—Very dissatisfied	0.004
				2—Dissatisfied	0.012
				3—Neutral	0.116
				4—Satisfied	0.349
				5—Very satisfied	0.519
Component 2: Patient-Centered User Experience (α = 0.848)					
Environment and privacy					
How comfortable were you with the screening location?	262	4.08	0.818	1—Very uncomfortable	0.019
				2—Uncomfortable	0.031
				3—Neutral	0.092
				4—Comfortable	0.573
				5—Very comfortable	0.286
How comfortable were you with the screening setting?	261	4.13	0.805	1—Very Uncomfortable	0.015
				2—Uncomfortable	0.031
				3—Neutral	0.080
				4—Comfortable	0.552
				5—Very comfortable	0.322
How comfortable were you with the privacy of the screening tool?	261	4.18	0.886	1—Very Uncomfortable	0.027
				2—Uncomfortable	0.031
				3—Neutral	0.065
				4—Comfortable	0.494
				5—Very comfortable	0.383

PESNS, Patient Experience Social Needs Screening; SD, standard deviation.

**Table 2.** Reliability of PESNS Questionnaire Factors

Item	Item–Total correlation^a^	Cronbach α^b^
How would you rate the screening tool’s ability to identify your health-related social needs?	0.529	0.827
How appropriate did you find the questions on the screening tool?	0.544	0.825
The understandability of the screening tool was:	0.438	0.835
How satisfied were you with the mode in which you completed the screening tool?	0.525	0.827
How would you describe the length of the screening tool?	0.488	0.833
Overall, how satisfied were you with the screening tool process?	0.669	0.812
How comfortable were you with the screening location?	0.547	0.825
How comfortable were you with the screening setting?	0.606	0.818
How comfortable were you with the privacy of the screening tool?	0.650	0.813

^a^ Corrected.

^b^ Cronbach’s α if the item is removed.

PESNS, Patient Experience Social Needs Screening.

PCA with varimax rotation produced an overall KMO of 0.839 and a significant Bartlett’s test of sphericity (*p* < 0.001), confirming data suitability for factor analysis. Table 3 displays the factor loadings: two factors with eigenvalues >1.0 were identified, accounting for 60% of the total variance. Factor 1, *Enhancing User Experience Essentials with Social Needs Screening in Health Care Settings*, included relevance, understandability, administration type and timing, and satisfaction (44.5% variance). Factor 2, *Patient-Centered User Experience of Social Needs Screening in Health Care Settings*, included environment and privacy (14.4% variance). PA/CFA analyses (Table 3) confirmed this structure, with all loadings exceeding 0.40.

**Table 3.** Combined Factor Loadings: PCA and PA/CFA Methods for PESNS Questionnaire

Item	PCA Component 1	PCA Component 2	PCA Communalities	PA/CFA Component 1	PA/CFA Component 2	PA/CFA Communalities
Relevance items						
Screening tool’s ability to identify HRSN	0.633	—	0.461	0.576	—	0.366
Appropriateness of questions	0.578	—	0.447	0.513	—	0.362
Understandability item						
Understandability	0.692	—	0.48	0.601	—	0.304
Administration type and timing items						
Satisfaction with mode	0.696	—	0.512	0.65	—	0.399
Perceived length	0.698		0.500	0.649		0.375
Satisfaction item						
Overall satisfaction	0.696	—	0.617	0.695	—	0.577
Environment and privacy items						
Comfort with location	—	0.886	0.797	—	0.912	0.712
Comfort with setting	—	0.873	0.799	—	0.861	0.721
Comfort with privacy	—	0.755	0.689	—	0.592	0.568
Percent of variance	44.5	14.4	—	44.5	14.4	—

Component loadings >0.50 (PCA) and >0.40 (PA/CFA) are considered significant.

PESNS, Patient Experience Social Needs Screening; PCA, principal component analysis; PA, principal axis; CFA, common factor analysis; HRSN, health-related social needs.

**FIG. 2.**
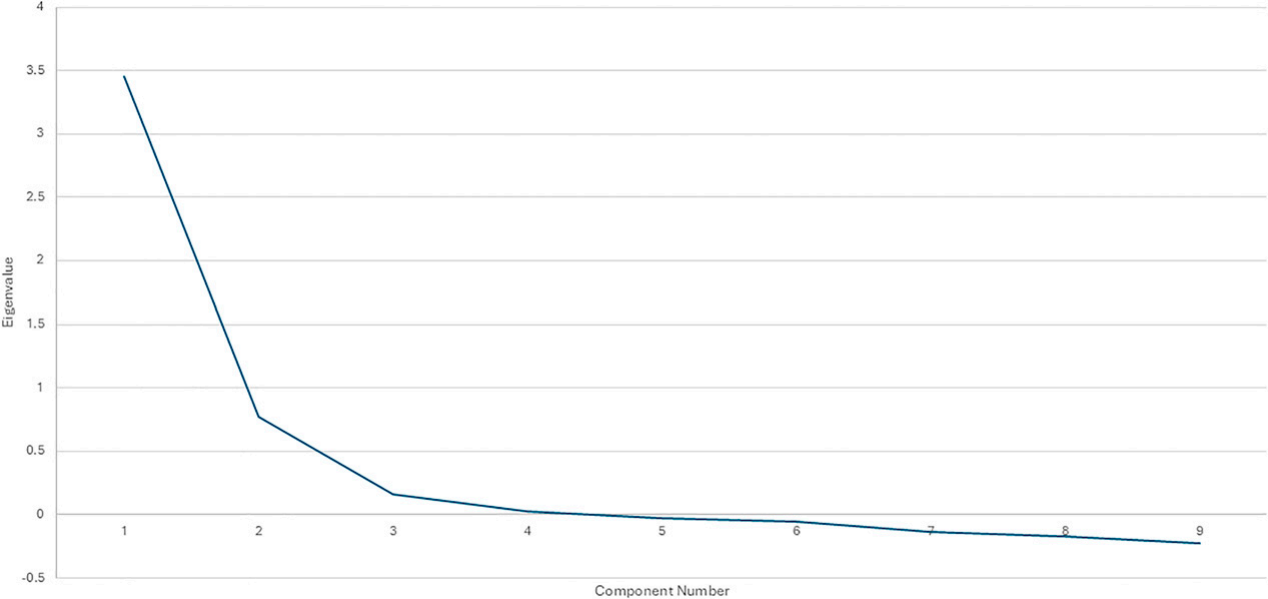
Scree plot showing eigenvalues for each component, in the factor extraction of data obtained from a PESNS questionnaire by 262 participants. PESNS, Patient Experience of Social Needs Screening.

Parallel analysis and scree plot (Fig. 2) confirmed the two-factor model, with the eigenvalues of 3.45 and 0.77, respectively. The polychoric correlation matrix (Table 4) revealed generally moderate associations among the items, supporting the validity of the PESNS questionnaire’s construct measurement.

**Table 4.** Polychoric Correlations Matrix PESNS Questionnaire

Items	How comfortable were you with the screening location?	How comfortable were you with the screening setting?	How comfortable were you with the privacy of the screening tool?	How would you rate the screening tool’s ability to identify your HRSN?	How appropriate did you find the questions on the screening tool?	The understandability of the screening tool was:	How satisfied were you with the mode in which you completed the screening tool?	How would you describe the length of the screening tool?	Overall, how satisfied were you with the screening tool process?
How comfortable were you with the screening location?	1.00	0.80	0.63	0.28	0.35	0.31	0.30	0.22	0.41
How comfortable were you with the screening setting?		1.00	0.70	0.32	0.38	0.27	0.40	0.35	0.59
How comfortable were you with the privacy of the screening tool?			1.00	0.51	0.47	0.32	0.47	0.39	0.57
How would you rate the screening tool’s ability to identify your HRSN?				1.00	0.54	0.40	0.45	0.40	0.54
How appropriate did you find the questions on the screening tool?					1.00	0.49	0.37	0.35	0.56
The understandability of the screening tool was:						1.00	0.48	0.41	0.45
How satisfied were you with the mode in which you completed the screening tool?							1.00	0.50	0.59
How would you describe the length of the screening tool?								1.00	0.64
Overall, how satisfied were you with the screening tool process?									1.00

## Discussion

A key strength of the 9-item PESNS questionnaire is its strong construct validity, as demonstrated by the two-factor structure identified through PCA and confirmed via CFA. Component 1 (Enhancing User Experience Essentials) captures the usability and effectiveness of the screening tool, whereas Component 2 (Patient-Centered User Experience) reflects the environmental and privacy-related aspects of the screening process. These findings align with prior research findings emphasizing that both tool functionality and clinical setting factors are critical determinants of patient engagement in social needs screenings.^36^

Specifically, Factor 1, with Cronbach’s α of 0.790, consisted of items to load onto PESNS Component 1, *Enhancing User Experience Essentials with Social Needs Screening in Health Care Settings*. The variable “Enhancing User Experience Essentials” could be clarified as the key factors or components that are essential for improving a user’s overall experience with a system, product, process, or service, for example, the patient’s overall experience with the clinical processes involved in screening for SDOH in health care settings. These essentials include factors such as the patient’s perceptions of the relevance, clarity, and timing of the social needs screening tool’s questions, ensuring that the language used is accessible and culturally appropriate. In addition, they involve optimizing the social needs screening administration workflow to enhance the overall patient experience and promote effective engagement during the screening process. The goal of this component is to create a seamless, intuitive, and engaging social needs screening experience for patients, addressing their needs efficiently while reducing frustration and enhancing overall usability. Improving the efficacy of these items could improve the patient response rates of SDOH screening in health care settings, particularly among targeted populations, for example, those participating in programs such as Medicare or Medicaid.

Prior studies have qualitatively explored the usability, functionality, and effectiveness of social needs screening processes to identify potential facilitators and barriers to participation in health care settings.^37,38^ For instance, a qualitative study involving primary care clinic staff in an underserved community identified barriers such as patient stigma about verbalizing social needs, provider frustration at eliciting needs they were unable to address, and provider unfamiliarity with community-based resources.^36^ Similarly, another study found that health care providers acknowledged the importance of assessing SDOH but faced challenges such as time constraints, perceptions of stigma, and limited referral protocols.^37^ These insights underscore the need for standardized, quantitative measures such as Component 1 of the PESNS questionnaire to effectively assess and address these factors in health care settings.

The high reliability of Component 1 of the PESNS questionnaire advances research by contributing a standardized, quantitative measure that provides a reliable framework for assessing key constructs related to the usability, functionality, and effectiveness of SDOH screening tools. This component evaluates critical factors such as the relevance, clarity, timing, and administration of screening questions as well as overall patient satisfaction with the process. By identifying both facilitators—such as the ease of understanding and appropriate administration methods—and barriers—such as unclear wording or ineffective workflows—Component 1 helps health care systems refine screening processes to enhance patient engagement and improve the integration of SDOH assessments into clinical care.

Factor 2, with Cronbach’s α of 0.848, consisted of items to load onto PESNS Component 2, *Patient-Centered User Experience of Social Needs Screening in Health Care Settings*. The variable “Patient-Centered User Experience” refers to the key elements necessary for enhancing the clinical structures of patient experience, such as the patient’s comfort with the location, setting, and privacy of social needs screenings in a health care environment. These factors typically include creating an environment where patients feel comfortable sharing details about their social challenges, particularly when they are unsure how this information will be used or if it will lead to meaningful support improving their overall health. The construct emphasizes optimizing these areas to ensure that patients are treated with dignity and respect, their privacy and confidentiality are maintained, and a clean, quiet, and well-coordinated care environment is provided to promote patient-centered care. Improving the effectiveness of these elements could help create an optimal patient experience, thereby enhancing response rates, especially among targeted populations participating in safety-net programs.

Patient-centered data practices hinge on understanding patients’ comfort with sharing their social needs information. Prior studies on the clinical structures of patient experience have highlighted the importance of comfort, privacy, and setting in shaping patient perceptions of screenings.^39^ Ensuring patient privacy during SDOH screenings is crucial, as breaches can lead to patient discomfort and reluctance to undergo necessary health care services. For instance, a study found that 10% of patients might refuse health care services due to privacy concerns, especially in acute care environments.^40^

The high reliability of PESNS questionnaire Component 2 adds to the literature by providing a standardized, quantitative measure that assesses how clinical structures—including the physical environment, privacy, and setting of social needs screenings—influence patient perceptions and experiences. This component evaluates key factors such as the comfort of the screening location (e.g., the waiting room/lobby, patient room, examination room, or hallway of an outpatient clinic, hospital, or ED), the privacy of the screening process (e.g., who was present and where it took place), and the overall patient-centered nature of the clinical environment. By capturing these dimensions, Component 2 helps identify structural facilitators and barriers that may impact patient trust, disclosure of social needs, and willingness to engage in screenings, offering actionable insights for health care systems to optimize SDOH screening conditions and improve patient-centered care.

### Applying health equity principles in health care settings

The distinction between Component 1 (screening usability) and Component 2 (screening environment) offers practical guidance for advancing health equity in health care settings. To promote equitable screening practices, interventions targeting Component 1 should refine the clarity, cultural accessibility, and ease of administration of social needs screening tools, ensuring that all patients—regardless of literacy, language, or background—can engage meaningfully. Efforts to strengthen Component 2 should focus on enhancing privacy, minimizing stigma, and creating respectful, welcoming physical environments that support the honest disclosure of social needs. Health care systems committed to health equity should use PESNS results as a patient-centered quality improvement metric to identify disparities in screening experiences and guide targeted improvements that ensure that social needs screenings are inclusive, dignified, and effective for diverse populations.

### Adapting the PESNS questionnaire for use with other validated SDOH screening tools

In health care settings, the PESNS questionnaire offers a structured approach to compare different SDOH screening tools, structures, and workflows, helping identify the optimal combination of factors shaping patient experience. The PESNS scale can be adapted and validated for use with various SDOH screening tools across different populations. For example, the item, “How comfortable were you with the privacy of the Accountable Health Communities Health-Related Social Needs Screening Tool?” can be modified by substituting the name with another standardized tool, such as PRAPARE. As social needs screenings become more common, understanding patient perceptions of screening questions and workflows will be essential for designing interventions that address SDOH effectively and promote health equity in diverse health care environments.

### Limitations and future directions

The limitations of this study should be considered when interpreting the findings. Although both components showed strong internal consistency, future research should assess test–retest reliability and predictive validity. Post-visit administration may introduce recall bias, and cross-cultural adaptation beyond English and Spanish is needed. Predictive relationships between PESNS scores and patient behaviors should be explored. Invariance analysis was not conducted; future studies should test measurement consistency across languages, demographics, and settings to strengthen the PESNS questionnaire’s validity and generalizability.

## Conclusion

The 9-item PESNS questionnaire demonstrates strong reliability and validity in measuring key dimensions of patients’ perceptions of SDOH screening tools and associated clinical workflows. It evaluates the tool’s relevance, understandability, administration type and timing, overall satisfaction, screening environment, and privacy. By integrating these constructs, the PESNS questionnaire provides a structured, patient-centered framework for identifying the facilitators and barriers to SDOH screening. It offers meaningful, data-driven insights that health care systems can use to refine workflows, improve patient engagement, advance health equity, and support continuous quality improvement in the implementation of social needs screenings.

## Abbreviations Used

AHC: Accountable Health Communities
CFA: Common factor analysis
CMS: Centers for Medicare & Medicaid Services
ED: Emergency department
EFA: Exploratory factor analysis
HRSN: Health-related social needs
KMO: Kaiser-Meyer-Olkin
PCA: Principal component analysis
PESNS: Patient Experience of Social Needs Screening
SDOH: Social determinants of health
